# The Use of Water to Relieve Pain

**Published:** 1877-04

**Authors:** 


The Use of Water to Relieve Pain.—The hypodermic use of water for relieving pain continues to afford an interesting object for experiment. The evidence in its favor could not be stronger, although little attempt is made to explain to us why or how water should quiet pain. Dr. Lafitte of Nantes has used water subcutaneously since 1872, when he succeeded in immediately relieving pain in a woman who was suffering most acutely from lumbago. Eight grmm. of distilled water was injected, and the pain did not return. In cases of sciatica, supra-orbital and facial neuralgia, as well as in intercostal neuralgia and rheumatic affections of the joints, he has found water injected subcutaneously quite as useful as morphia. Dr. Pillet speaks highly of hypodermic injections of water in lumbago and intercostal neuralgia. Dr. Lelut says that for the last three months he has used the pure water injections, with the best results. He relates how he came to use it. His servant one day upset the bottle containing his morphia solution for subcutaneous injections, and, to conceal her clumsiness, filled the bottle with ordinary water. Dr. Lelut, not knowing this, injected the water into the thigh of a patient who was suffering severely from sciatica, and whom he was treating by the subcutaneous injection of morphia. The patient was astonished at the instant relief of the pain, and said: "What kind of a liquid is this you are using which causes me no uneasiness or no sickness at the stomach like the former?" Since then Dr. Lelut has used nothing subcutaneously but water.

Dr. Dresch praises the usefulness of this injection, especially in muscular rheumatism. He also tells of a case of osteo-sarcoma of the thigh, in which he used daily 60 ctgm. of morphia subcutaneously, chloral, cicuta and other remedies, and where hypodermic injections of water succeeded in relieving the pain quite as well as morphia, without producing the disagreeable constitutional effects of that drug. Dr. Dresch does not use simple water, but prefers peppermint water.

Dr. Burney Yeo, of London, says he found subcutaneous injections of water useful in relieving the pain of a patient suffering from thoracic aneurism.—*Western Lancet.*

